# Protective Antibodies against Placental Malaria and Poor Outcomes during Pregnancy, Benin

**DOI:** 10.3201/eid2105.141626

**Published:** 2015-05

**Authors:** Nicaise Tuikue Ndam, Lise Denoeud-Ndam, Justin Doritchamou, Firmine Viwami, Ali Salanti, Morten A. Nielsen, Nadine Fievet, Achille Massougbodji, Adrian J.F. Luty, Philippe Deloron

**Affiliations:** Institut de Recherche pour le Développement, Paris, France (N. Tuikue Ndam, L. Denoeud-Ndam, J. Doritchamou, N. Fievet, A.J.F. Luty, P. Deloron);; Pôle de Recherche et d’Enseignement Supérieur Sorbonne Paris Cité, Paris (N.Tuikue Ndam, N. Fievet, A.J.F. Luty, P. Deloron),; Université d’Abomey-Calavi, Cotonou, Benin (F. Viwami, A. Massougbodji, A.J.F. Luty);; University of Copenhagen, Copenhagen, Denmark (A. Salanti, M.A. Nielsen)

**Keywords:** Plasmodium falciparum, parasites, malaria, placental malaria, VAR2CSA, antibodies, erythrocytes, pregnancy, outcomes, Benin

## Abstract

Placental malaria is caused by *Plasmodium falciparum*–infected erythrocytes that bind to placental tissue. Binding is mediated by VAR2CSA, a parasite antigen coded by the *var* gene, which interacts with chondroitin sulfate A (CSA). Consequences include maternal anemia and fetal growth retardation. Antibody-mediated immunity to placental malaria is acquired during successive pregnancies, but the target of VAR2CSA-specific protective antibodies is unclear. We assessed VAR2CSA-specific antibodies in pregnant women and analyzed their relationships with protection against placental infection, preterm birth, and low birthweight. Antibody responses to the N-terminal region of VAR2CSA during early pregnancy were associated with reduced risks for infections and low birthweight. Among women infected during pregnancy, an increase in CSA binding inhibition was associated with reduced risks for placental infection, preterm birth, and low birthweight. These data suggest that antibodies against VAR2CSA N-terminal region mediate immunity to placental malaria and associated outcomes. Our results validate current vaccine development efforts with VAR2CSA N-terminal constructs.

## Abstract 2

Immunity requires a vaccine that inhibits binding of infected erythrocytes to chondroitin sulfate.

Tissue sequestration of *Plasmodium falciparum*–infected erythrocytes drives malaria-related pathologic changes (*1*). Tissue sequestration is primarily mediated by members of the parasite variant antigen family of *P. falciparum* erythrocyte membrane protein 1, which is expressed on the membrane of infected erythrocytes. These proteins display extensive antigenic variation, concurrently changing receptor recognition, and tissue tropism of infected erythrocytes (*2*). Accumulation of infected erythrocytes in placental intervillous spaces characterizes malaria during pregnancy (*3*). This sequestration of infected erythrocytes results in maternal anemia and low birthweight (LBW) (*4*–*6*), as well as consequences for child health (*7*–*10*).

Sequestration of infected erythrocytes in the placenta is mediated by VAR2CSA, the *P. falciparum* erythrocyte membrane protein 1 variant that binds to chondroitin sulfate A (CSA) on the syncytiotrophoblast (*11*,*12*). VAR2CSA is a multidomain protein (≈350 kDa). Acquisition of antibodies against VAR2CSA occurs during pregnancy after exposure to infected erythrocytes sequestering in the placenta. Concentrations of these antibodies and those of antibodies that inhibit binding of infected erythrocytes to CSA (*13*,*14*) increase with parity. Furthermore, women with VAR2CSA-specific antibodies give birth to babies with higher birthweights (*15*). VAR2CSA-expressing parasites are the primary cause of placental malaria (*16**,**17*), which suggests that parasites can escape preexisting immunity (i.e., that naturally acquired immunity against preerythrocytic or erythrocytic stages of malaria does not protect against this syndrome).

The demonstration that parasites that have the *var2csa* knockout gene irreversibly lose the ability to adhere to CSA (*18*), as well as the ability of VAR2CSA to induce antibodies that inhibit adherence of placental infective erythrocytes to CSA in vitro, strongly argue for use of VAR2CSA as a vaccine against placental malaria. However, VAR2CSA-based vaccine research is challenged by the size and polymorphism of this protein and requires identification of smaller functional domains that combine an ability to induce strain-transcending antibody responses with a facility of production in a recombinant protein form. Therefore, identifying the region of VAR2CSA that induces antibodies associated with protection in multigravid women in malaria-endemic regions is a priority. The VAR2CSA critical CSA binding site is located in its N-terminal region (*19*–*21*), but the characteristics of naturally acquired antibodies against this region remain to be defined.

The Strategies to Prevent Pregnancy-associated Malaria Project, a cohort study of pregnant women enrolled early in pregnancy and followed up until delivery, was conducted during 2008–2011 in Comé in southern Benin. In this substudy, we assessed the effect of antibody response to placental infected erythrocytes, measured early in pregnancy and at delivery, on major pregnancy outcomes.

## Materials and Methods

### Study Site and Population

A detailed description of the Comé area has been reported (*22*). The Strategies to Prevent Pregnancy-associated Malaria Project was approved by the Comité Consultatif de Déontologie et d’Ethique of the Research Institute for Development (Paris, France) and the ethical committee of the Faculty of Health Sciences (University of Abomey-Calavi, Cotonou, Benin). Pregnant women were provided information about the study at the first antenatal visit during their first or second trimester of pregnancy (gestational age <24 weeks).

Women from whom informed consent was obtained were included in the study and followed up until delivery. A clinical examination was conducted, and 10 mL of venous blood was collected at inclusion and during each antenatal or emergency visit. Ultrasonography was performed by using a portable ultrasound system (Titan Ultrasound System; SonoSite Inc., Bothell, WA, USA) to determine exact gestational age and to plot fetal growth. Fetal growth alterations were used to define children born small-for-gestational age (SGA) (*23*). At delivery, peripheral and perfused placental blood samples were collected along with clinical data for the newborn.

Women were given 2 doses of intermittent preventive treatment in pregnancy with sulfadoxine/pyrimethamine (IPTp-SP) (Stichting International Dispensary Foundation, Amsterdam, the Netherlands) at least 1 month apart in the second–third trimesters of pregnancy under supervision of midwives according to national guidelines in Benin. When women had clinical symptoms between antenatal visits, they were encouraged to report to health facilities. Any participant with fever (axillary temperature >37.5°C) and malaria, as assessed by a rapid diagnostic test, received quinine or SP if this treatment coincided with scheduled IPTp intake.

### Diagnosis of Infection with *P. falciparum*

At each visit, a rapid diagnostic test for *P. falciparum* was performed, and thick and thin blood smears were prepared and double-read according to standard procedures. At delivery, blood smears were prepared from placental blood.

### Plasma Antibody against *P. falciparum*–Infected Erythrocyte Surface

Plasma samples collected at inclusion and delivery were analyzed by using *P.* *falciparum* strain FCR3. Parasite cultures were selected by panning (enriching) on BeWo cells as described (*24*). The ability of plasma to label the surface of late-stage infected erythrocytes was tested as described (*25**,**26*). Antibody surface-labeling of ethidium bromide–positive infected erythrocytes was quantified by using flow cytometry, and data were analyzed by using CellQuest Pro or FlowJo version 7.6 (TreeStar, Ashland, OR, USA). Median fluorescence intensity was converted into relative fluorescence intensity as described (*27*).

### Antibody-Mediated Inhibition of Infected Erythrocyte Adherence to Chondroitin Sulfate Proteoglycan

For an inhibition of binding assay (IBA), a petri dish was coated overnight with phosphate-buffered saline (PBS) containing 1% bovine serum albumin (BSA) and 5 μg/mL decorin (chondroitin sulfate proteoglycan [CSPG]; Sigma, St. Louis, MO, USA) and blocked with 3% BSA in PBS for 30 min. Late-stage FCR3 Bewo-selected infected erythrocytes were blocked in BSA/RPMI medium for 30 min. A 20% parasite suspension was incubated with plasma (1:5 dilution) or 500 μg/mL soluble CSA for 30 min at room temperature, added to the ligand, and incubated for 15 min at room temperature (*28*). Nonadherent cells were removed by using an automated washing system. Cells were fixed with 1.5% glutaraldehyde in PBS and stained with Giemsa. Adherent infected erythrocytes were quantified by microscopy as number of infected erythrocytes bound per square milliliter (*16*).

### Plasma Antibody Levels against Recombinant *P. falciparum* VAR2CSA

The full-length ectodomain of VAR2CSA (FV2) from the FCR3 strain and the truncation corresponding to Duffy binding-like (DBL) antigen (DBL1–DBL2 encompassing 2 domains, DBL3, DBL4, DBL5, and DBL6 domains) were produced in baculovirus-infected SF9 cells as described (*11*,*19*,*29*). Recombinant protein of *P. falciparum* apical membrane antigen 1 (PfAMA1) from the FVO strain was also used.

Levels of specific IgG against VAR2CSA were measured in plasma samples by using an ELISA as described (*13*). In brief, microtiter plates were coated with 0.5 μg/mL of each protein and incubated overnight at 4°C with 100 μL of plasma at dilutions of 1:100 (for antibodies against VAR2CSA) or 1:1,000 (for antibodies against PfAMA1). Plates were washed 3 times with 0.1% PBS-Tween 20, and a 1:15,000 dilution of horseradish peroxidase–conjugated antibody against human IgG (Sigma-Aldrich, St. Louis, MO, USA) was added to each well and incubated at room temperature for 1 h. After plates were washed 4 times, antibody reactivity was visualized at 450 nm after addition of tetramethylbenzidine (Sigma-Aldrich). The negative control pool consisted of plasma samples from pregnant women in France who had no history of travel to malaria-endemic areas. The positive control pool consisted of plasma samples from multigravid women from Benin who had known high levels of surface reactivity to infected erythrocytes from placental isolates. Optical density values were converted into arbitrary absorbance units as described (*30*). Threshold of positivity was defined for each antigen from the mean ± 3 SD response of 30 unexposed pregnant women from France.

### Statistical Analysis

Categorical variables were compared by using the Fisher exact test. Comparisons between groups were made by using nonparametric tests (Kruskal-Wallis test for unpaired comparisons and paired Wilcoxon test to compare levels between inclusion and delivery within the same persons). Correlations between antibody levels and different antigens and with binding inhibition capacity of plasma were studied by using the Spearman rank correlation test.

For association between antibody levels and protection against infection or poor birth outcomes, we first considered antibody levels in plasma samples at inclusion to address a cause–effect chronology. Association between antibody levels was sought with key pregnancy outcomes, including number of peripheral *P. falciparum* infections, placental infection, LBW, maternal anemia at delivery, and preterm birth (PTB). Multivariate logistic regression modeled the effect of each antibody (defined in quartiles) on the outcome after adjustment for study center, gravidity (primigravidae versus multigravidae), and *P. falciparum* infection at inclusion.

To study the effect of antibody levels early in pregnancy on the number of infections occurring during the follow-up period, we adjusted a binomial negative model for the same covariates and offset by the duration of the follow-up period. The binomial negative distribution was used instead of a Poisson distribution to account for data overdispersion. In all models, interaction between infection at inclusion and antibody levels was tested, and results were stratified when appropriate. Type 1 error for significance was 0.05. To account for multiple testing, we applied the Holm-Bonferroni method (*31*); corrected p values are given when necessary.

Given the potential for antibody maturation during the follow-up period and a subsequent increase in specificity, association between increases in plasma binding inhibitory capacity and protection against infection was also analyzed at delivery. The same modeling approach was used with placental infection, LBW, PTB, and SGA in an appropriate subgroup of women (i.e., women with >1 documented infection during the follow-up period were classified as truly exposed).

## Results

### Study Profile and Population

The study site and population have been reported (*22*). In brief, 854 women took both doses of IPTp-SP and were followed up until delivery. One fourth of them slept under bed nets. Antibody assays were performed with samples from the 710 women for whom clinical data and plasma samples were available. A total of 326 (46%) women had >1 parasitemia throughout the follow-up period, including 116 (16%) women who were infected at inclusion. Eighty-two women who had fever received curative treatment with quinine, and 6 received other antimalarial drugs. A total of 546 microscopically detectable parasitemias were recorded. At delivery, placental infection was observed in 70 (12%) women; this infection was the only parasitemia recorded for 17 women. Prevalence of placental infection was highest in primigravid women (19%) and decreased to 12% in those with second or third pregnancies and to 9% in those with fourth or more pregnancies (p = 0.025, by Fisher exact test). Mean (± SD) birthweight was 3,002 (486) g. A total of 10% of the babies had LBW, and 8% had PTB (Table 1).

**Table 1:** Characteristics of 710 women and their infants in study of protective antibodies against placental malaria and poor outcomes during pregnancy, Benin*

Characteristic	Value
At enrollment	
Study center, no. (%)	
Akodeha	279 (39)
Comé	266 (38)
Wedeme Pedah	165 (23)
Age, y, mean ± SD, n = 698	26.7 ± 6.3
Gravidity, no. (%)	
Primigravidae	115 (16)
Secundigravidae	154 (22)
Multigravidae	441 (62)
Gestational age, wk, mean ± SD	16.6 ± 4.8
HIV status	
Positive, no. (%)	13 (1.8)
Negative, no. (%)	697 (98.2)
Hb level, g/dL, mean ± SD, n = 704	10.6 ± 1.3
Anemia (Hb level <11 g/dL), no. (%), n = 704	439 (62)
Malaria infection, no. (%)†	116 (16)
During follow-up	
No. antenatal visits, median (IQR)	5 (4–6)
Anemia during follow-up, no. (%), n = 708	619 (87)
No. malaria infections during follow-up, no. (%)†	
0	384 (54)
1	183 (26)
2	89 (12)
>3	54 (8)
Treatment for malaria other than IPTp-SP, no. (%)‡	88 (12)
At delivery	
Twin delivery, no. (%)	16 (2)
Birthweight, g, mean ± SD, n = 679§	3,002 ± 486
Low birthweight infant (<2,500 g), no. (%), n = 679§	71 (10)
Gestational age, wk, mean ± SD, n = 680§	39.6 ± 2.0
Small for gestational age, no. (%), n = 612§	100 (16)
Preterm birth (age <37 wk), no. (%), n = 680§	53 (8)
Hb level, g/dL, mean ± SD, n = 649	11.0 ± 1.4
Anemia (Hb level <11 g/dL), no. (%), n = 649	289 (45)
Placental malaria detected by blood smear, no. (%), n = 60	70 (12)

*Hb, hemoglobin; IQR, interquartile range; IPTp-SP, intermittent preventive treatment in pregnancy with sulfadoxine/pyrimethamine. †Defined by a positive blood smear regardless of symptoms. ‡82 women received quinine and 6 received other antimalarial medications. §Excluding twins and stillbirths.

### Modification of Acquisition of VAR2CSA-Specific IgG during Pregnancy by *P. falciparum* Infection

**Figure 1:**
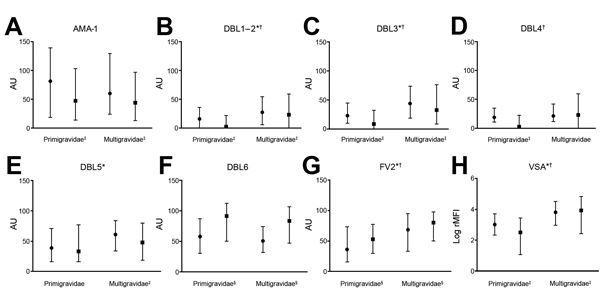
Antibody levels at study inclusion and delivery, by parity, against placental malaria in pregnant women, Benin. A) Apical membrane antigen 1 (AMA-1); B–F) Duffy binding-like (DBL) antigen; G) Full-length ectodomain of variant surface antigen 2 chondroitin sulfate (FV2); H) Variant surface antigen (VSA). Solid circles indicate medians for inclusion, solid squares indicate medians for delivery, and error bars indicate interquartile ranges. AU, absorbance units; rMFI, relative median fluorescence intensity. *Parity dependence at inclusion (p<0.05 by Fisher exact test). †Parity dependence at delivery (p<0.05 by Fisher exact test). ‡Decrease between inclusion and delivery (p<0.05 by paired Wilcoxon test). §Increase between inclusion and delivery (p<0.05 by paired Wilcoxon test).

All 6 recombinant VAR2CSA proteins were detected by ELISA in plasma samples from pregnant women (Figure 1). Specific antibodies were present at high levels at inclusion and delivery, and responses to the 6 VAR2CSA recombinant proteins were correlated with each other (0.28<r<0.77, p<0.0001 for all comparisons). Between inclusion and delivery, responses to all VAR2CSA proteins decreased, except for those to DBL6 and the full-length construct (FV2). The IPTp-SP that women received effectively reduced contact with blood-stage parasites.

**Figure 2:**
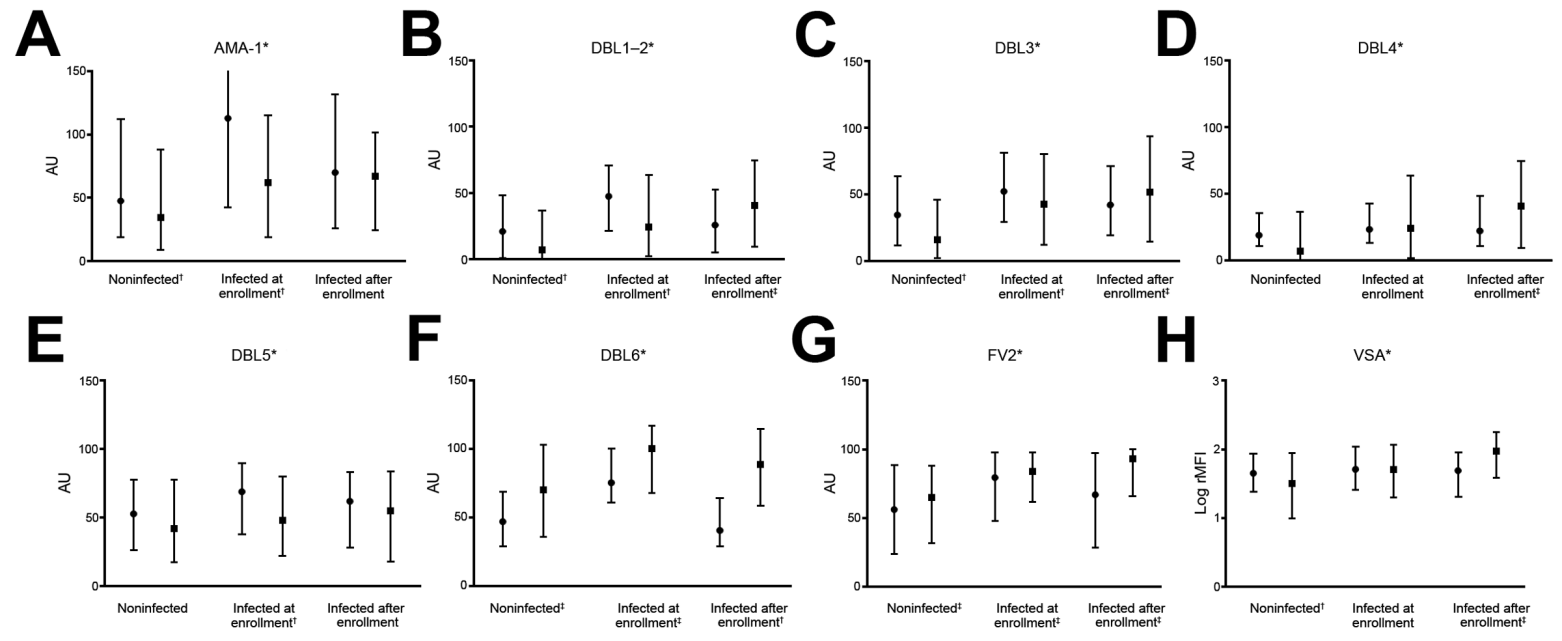
Antibody levels at study inclusion and delivery, by parasitemia during pregnancy, against placental malaria in pregnant women, Benin. A) Apical membrane antigen 1 (AMA-1); B–F) Duffy binding-like (DBL) antigen; G) Full-length ectodomain of variant surface antigen 2 chondroitin sulfate (FV2); H) Variant surface antigen (VSA). Solid circles indicate medians for inclusion, solid squares indicate medians for delivery, and error bars indicate interquartile ranges, and error bars indicate interquartile ranges. AU, absorbance units; rMFI, relative median fluorescence intensity. *Significantly higher in women with parasitemia during pregnancy (p<0.05 by Fisher exact test). †Decrease between inclusion and delivery (p<0.05 by paired Wilcoxon test). ‡Increase between inclusion and delivery (p<0.05 by paired Wilcoxon test).

Women were assigned to 2 subgroups: those who had >1 parasitemia during the follow-up period and those who did not (Figure 2). At delivery, IgG responses to all VAR2CSA proteins were higher for women infected during follow-up period than in the other women. In infected women, antibody responses between inclusion and delivery increased (p<0.001 for all comparisons) or were unchanged (DBL5 and PfAMA-1). Conversely, for women who were not infected, antibody levels decreased, except those against DBL6 and FV2 (Figure 2). Women infected at inclusion (at blood sampling) had higher antibody responses to all VAR2CSA proteins than those who were uninfected.

### Effect of Gravidity on VAR2CSA-Specific Antibody Levels

Antibody responses to VAR2CSA proteins other than DBL4 and DBL6 increased with gravidity. Plasma levels of antibodies against VSA (reactive with erythrocyte surface) showed similar profiles of gravidity dependence at inclusion and at delivery (Figure 1). Proportions of women seropositive for different antigens at inclusion and delivery are shown in Table 2. Relationships with gravidity remained for all proteins except for DBL6.

**Table 2:** Percentage of antibody responders, by parity, in study of protective antibodies against placental malaria and poor outcomes during pregnancy, Benin*

Antigen or assay	Enrollment				Delivery			
	All, n = 710	Primigravidae, n = 115	Multigravidae, n = 595	p value	All, n = 710	Primigravidae, n = 115	Multigravidae, n = 595	p value
AMA1	77	73	78	0.265	69	70	69	0.732
VSA	67	46	71	<0.0001	58	32	63	<0.0001
DBL1–DBL2	53	41	56	0.005	46	25	50	<0.0001
DBL3	65	45	69	<0.0001	51	28	55	<0.0001
DBL4	42	40	43	0.569	46	26	50	0.002
DBL5	71	55	74	<0.0001	58	49	60	0.035
DBL6	67	70	66	0.607	81	85	80	0.434
FV2	71	51	75	<0.0001	81	70	83	0.004
IBA	59	42	62	<0.0001	66	61	67	0.168

*Antibody responders were defined as persons having an antibody level beyond the threshold. The threshold was defined for each antigen as mean reactivity + 3 SD for 30 unexposed French pregnant women. AMA1, apical membrane antigen 1; VSA, variant surface antigen; DBL, Duffy binding-like antigen; FV2, full-length ectodomain of variant surface antigen 2 chondroitin sulfate; IBA, inhibition of binding assay.

### Antibody Levels at Inclusion and Association with Protection against Infection

Antibodies were tested in separate models after adjustment for study site, gravidity, and infection at inclusion. Results are summarized in Table 3. We first investigated the relationship between antibody responses at inclusion (divided into quartiles) and number of infections during the follow-up period by using binomial negative regression modeling. The independent variable was the number of infections during the follow-up period, excluding infection at inclusion, which was used as an adjustment covariate. High responses to particular VAR2CSA antigens (FV2, DBL3X) at inclusion were associated with a lower subsequent risk for *P. falciparum* parasitemia.

**Table 3:** Association of antibody levels at enrollment with pregnancy-associated malaria and pregnancy outcomes in 710 pregnant women, Benin*

Characteristic	No malaria infections/mo		Placental malaria		Low birthweight	
	IRR (95% CI)	p value	OR (95% CI)	p value	OR (95% CI)	p value
VSA, range log RMFI	n = 709	0.288	n = 607	0.022†	n = 692	0.721
Second quartile, 2.8–3.7	0.85 (0.55–1.31)†		0.58 (0.28–1.18)		1.25 (0.65–2.43)	
Third quartile, 3.7–4.4	0.84 (0.54–1.30)†		0.43 (0.21–0.91)†		0.84 (0.41–1.75)	
Higher quartile, >4.4	1.14 (0.75–1.74)		0.40 (0.19–0.85)†		1.05 (0.51–2.17)	
VAR2CSA DBL1–DBL2, AU	n = 709	0.223†	n = 607	0.61	n = 692	**0.003**†
Second quartile, 4–26	0.79 (0.51–1.24)†		1.07 (0.49–2.36)		0.71 (0.39–1.30)	
Third quartile, 26–54	0.74 (0.47–1.17)†		1.46 (0.69–3.05)		0.36 (0.17–0.74)†	
Higher quartile, >54	1.01 (0.64–1.60)		0.95 (0.43–2.13)		0.33 (0.16–0.71)†	
VAR2CSA DBL3, AU	n = 706	**0.0001**†	n = 604	**0.011**†	n = 689	0.0236
Second quartile, 17–40	0.43 (0.27–0.70)†		0.45 (0.21–0.99)†		0.54 (0.28–1.05)	
Third quartile, 0–70	0.45 (0.27–0.75)†		0.41 (0.19–0.90)†		0.29 (0.13–0.65)	
Higher quartile, >70	0.80 (0.48–1.32)		0.40 (0.18–0.92)†		0.50 (0.24–1.07)	
VAR2CSA DBL4, AU	n = 703	0.122†	n = 601	0.69	n = 686	0.352†
Second quartile, 11–21	0.69 (0.44–1.07)†		0.84 (0.40–1.78)		0.83 (0.44–1.57)	
Third quartile, 21–40	0.71 (0.45–1.08)†		0.70 (0.32–1.53)		0.67 (0.34–1.32)†	
Higher quartile, >40	0.88 (0.57–1.36)		1.07 (0.52–2.22)		0.63 (0.31–1.30)†	
VAR2CSA DBL5, AU	n = 705	0.019	n = 603	0.79	n = 688	0.675
Second quartile, 29–56	0.67 (0.43–1.04)		1.15 (0.54–2.44)		1.39 (0.70–2.75)	
Third quartile, 59–84	0.82 (0.52–1.29)		0.98 (0.45–2.14)		1.25 (0.60–2.62)	
Higher quartile, >84	1.30 (0.83–2.05)		1.31 (0.60–2.84)		1.54 (0.75–3.14)	
VAR2CSA DBL6, AU	n = 352		n = 306		n = 343	0.118
Second quartile, 32–52	0.94 (0.51–1.73)	0.96	0.56 (0.20–1.56)	0.21	0.25 (0.08–0.79)	
Third quartile, 52–74	0.84 (0.44–1.60)		0.29 (0.09–0.93)		0.83 (0.36–1.96)	
Higher quartile, >74	0.95 (0.50–1.83)		0.47 (0.17–1.36)		0.65 (0.26–1.60)	
FV2, AU	n = 698		n = 596		n = 681	
Second quartile, 29–65	0.83 (0.53–1.29)	**0.0005**	1.39 (0.66–2.93)	0.34	0.80 (0.42–1.54)	0.55
Third quartile, 65–94	0.62 (0.39–0.99)		0.74 (0.32–1.69)		0.58 (0.28–1.21)	
Higher quartile, >94	1.52 (0.97–2.39)		0.84 (0.37–1.93)		0.82 (0.39–1.71)	
IBA, % inhibition	n = 703	0.226†	n = 602	0.65	n = 686	0.21†
Second quartile, 25–40	0.93 (0.59–1.45)		0.83 (0.39–1.76)		1.06 (0.57–1.97)	
Third quartile, 40–60	1.30 (0.84–2.01)†		0.98 (0.46–2.09)		0.68 (0.34–1.37)†	
Higher quartile, >60	1.21 (0.77–1.90)†		1.34 (0.64–2.82)		0.62 (0.30–1.29)†	
AMA1, AU	n = 706	0.463	n = 604	0.58	n = 689	0.155†
Second quartile, 23–61	0.98 (0.62–1.55)		0.78 (0.37–1.65)		0.71 (0.36–1.42)†	
Third quartile, 61–131	0.76 (0.48–1.20)		0.58 (0.27–1.25)		0.68 (0.35–1.33)†	
Higher quartile, >131	1.04 (0.65–1.65)		0.73 (0.35–1.50)		0.64 (0.32–1.27)†	

*Antibodies were tested individually after adjustment for study site, rank of gestation, and malaria at enrollment. p values were obtained by using the Wald test. Bold indicates significant p values after the Holm-Bonferroni correction was used for multiple testing. Analysis of placental malaria was restricted to 607 women who reached delivery, had antibody measurements, and had a placental blood smear. Analysis of low birthweight was restricted to 692 women who reached delivery and had an evaluation of birthweight. IRR, incidence rate ratio; OR, odds ratio; VSA, variant surface antigen; RMFI, relative mean fluorescence intensity; VAR2CSA, variant surface antigen 2 chondroitin sulfate; DBL, Duffy binding-like antigen; AU, absorbance units; FV2, full-length ectodomain of variant surface antigen 2 chondroitin sulfate; IBA, inhibition of binding assay; AMA1, apical membrane antigen 1. †When ORs for 2 contiguous quartiles were similar, p values were computed after grouping those quartiles.

We then tested whether antibody responses at inclusion were predictive of placental infection at delivery by using multivariate logistic regression. High responses to DBL3X at inclusion were associated with reduced prevalence of placental infection at delivery (p = 0.011). A trend between strong antibody responses against VSA and placental infection was observed.

We investigated relationships between antibody responses at inclusion and LBW. Strong IgG responses against DBL1–DBL2 were associated with reduced prevalence of LBW babies (p = 0.003); a similar trend was observed for responses to DBL3X (p = 0.02) (Table 3). High levels of these antibodies were also associated with increased mean birthweight. In addition, high levels of DBL3X-specific antibodies showed a trend toward protection against being born with SGA (p = 0.013). No relationships were observed for PTB or maternal anemia at delivery.

### Effect of Gravidity and Pregnancy-Associated *P. falciparum* Infections on Plasma to Inhibit Binding of Infected Erythrocytes to CSPG

**Figure 3:**
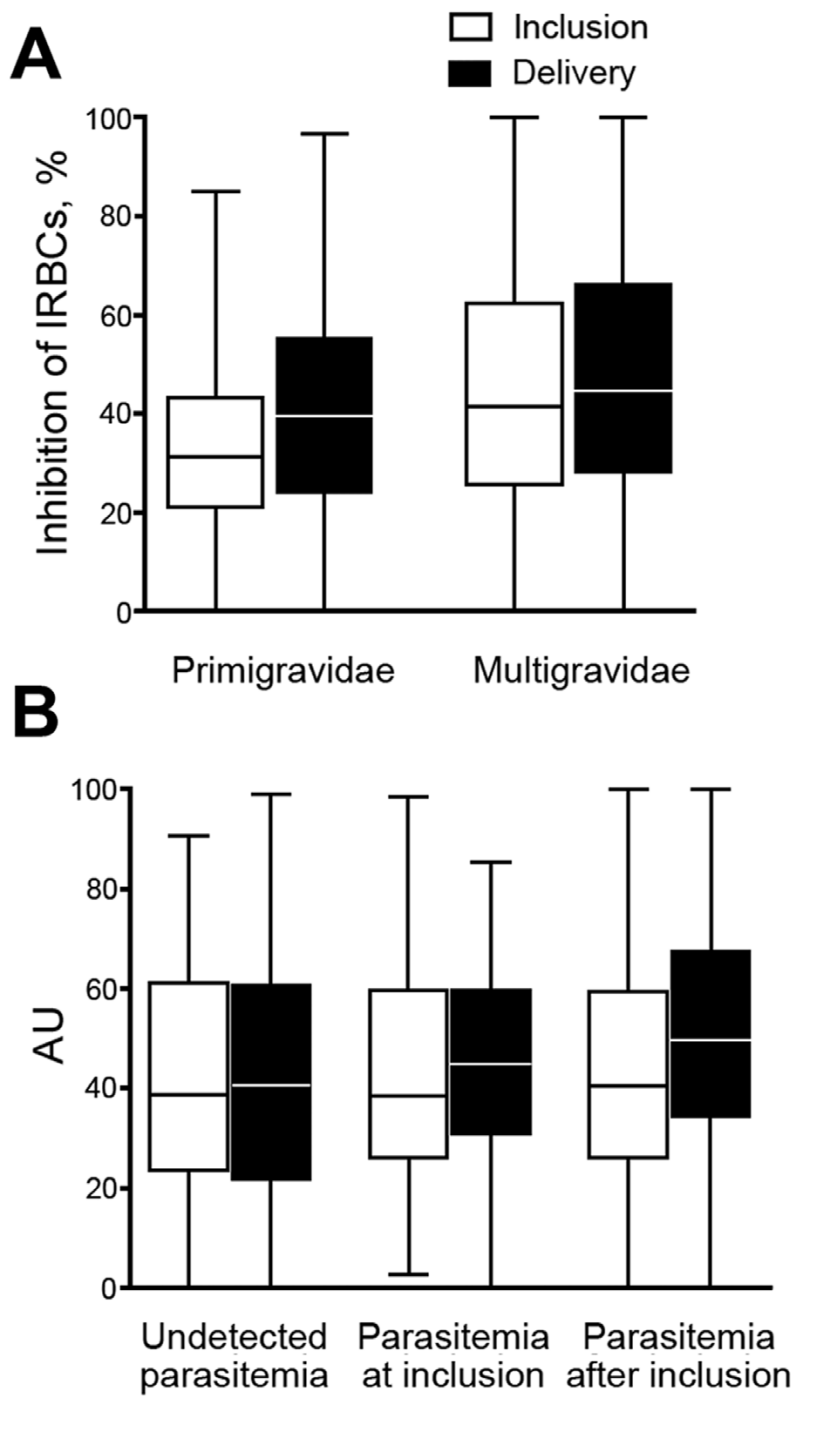
Binding inhibition profile of plasma from pregnant women against placental malaria, Benin. Plasma binding inhibitory capacity according to parity (n = 109 primigravidae and 573 multigravidae) (A) and to parasitemia during follow-up (B) (n = 384 women with undetected parasitemia, 115 with parasitemia detected at study inclusion, and 183 with parasitemia detected after inclusion). A) Binding inhibitory capacity was significantly higher at inclusion in multigravidae than in primigravidae and increased at delivery compared with that at inclusion in both groups (all p<0.05). B) Significant increase between inclusion and delivery and a higher level at delivery in women with documented parasitemia during pregnancy (p<0.05, by Fisher exact test). Horizontal lines indicate medians, boxes indicate interquartile ranges, and error bars indicate ranges. IRBCs, infected red blood cells; AU, absorbance units.

The ability of plasma samples to inhibit infected erythrocyte binding to CSPG was higher for multigravidae than for primigravidae at inclusion (p<0.001) and delivery (p<0.008) (Figure 3). Unlike antibodies measured by ELISA and fluorescent-activated cell sorting, the binding inhibitory capacity increased between inclusion and delivery (Figure 3, panel A) for primigravidae (p = 0.006) and multigravidae (p<0.001). When women were divided into subgroups according to infection history, the inhibitory capacity increased between inclusion and delivery only among women who were infected at least once (p<0.001) (Figure 3, panel B).

### Binding Inhibitory Capacity of Plasma and Pregnancy Outcomes

**Figure 4:**
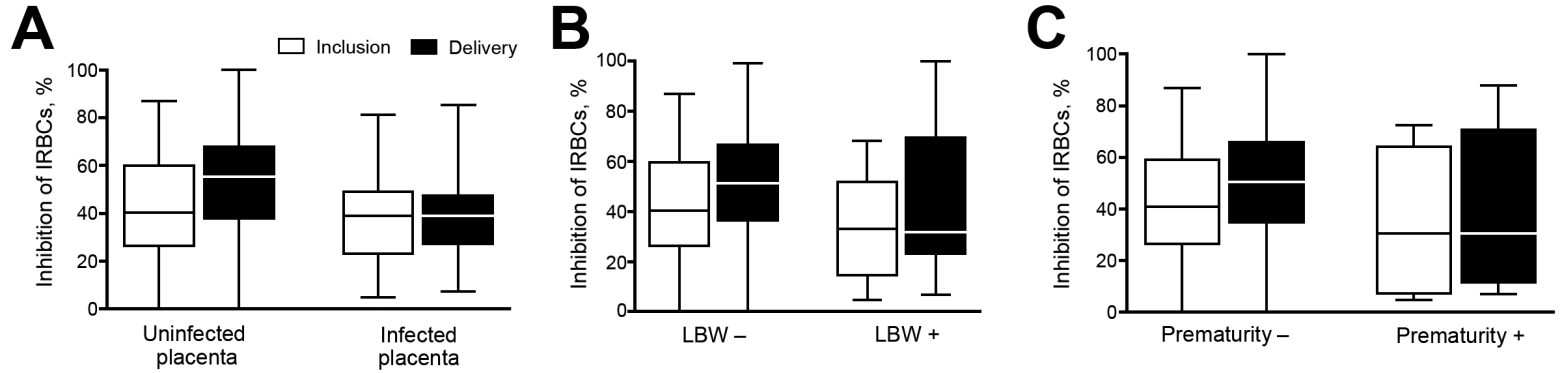
Binding inhibitory capacity of plasma, by adverse outcomes, in pregnant women with documented *Plasmodium falciparum* infection during follow-up, Benin. Binding inhibition was assessed according to adverse outcomes in the subgroup of women who had ≥1 parasitemia documented between study inclusion and delivery. A) Placental infection (52 infected placentas and 214 uninfected placentas). B) Low birthweight (LBW) (36 with LBW and 254 without LBW). C) Preterm birth (29 preterm and 269 not preterm). Horizontal lines indicate medians, boxes indicate interquartile ranges, and error bars indicate ranges. Plasma binding inhibitory capacity was significantly higher at delivery in women without adverse outcomes (p<0.05, by Fisher exact test), and the increase between inclusion and delivery was also significant (p<0.05, by paired-Wilcoxon test). No associations were observed at inclusion. IRBCs, infected red blood cells.

The level of IBA at inclusion was not associated with protection from adverse pregnancy outcomes (Table 3). Because IBA levels increased at delivery for women with infection during the follow-up period, we investigated the relationship between IBA levels at delivery and protection from poor outcomes. Among the 309 women infected at least once before delivery, an increase in plasma IBA activity between inclusion and delivery was associated with absence of placental infection at delivery (Figure 4, panel A), absence of LBW (Figure 4, panel B), and absence of PTB (Figure 4, panel C) (p<0.0001 for all comparisons).

Multivariate logistic regression models confirmed the protection conferred by IBA capacity at delivery against adverse outcomes of pregnancy (Table 4). Women with higher binding inhibitory capacity at delivery were less likely to deliver LBW babies (odds ratio <1 for all quartiles in comparison to first quartile; p = 0.028). There was a stronger relationship with protection from SGA (p = 0.0084). Because of a major interaction term, we divided women into subgroups according to the presence or absence of infection at inclusion. For women not infected at inclusion, higher IBA capacity at delivery was associated with a lower risk for placental malaria (p = 0.008) and PTB (p = 0.032), but these associations were not evident for those infected at inclusion (Figure 3, panel B; Table 4).

**Table 4:** Association between inhibition capacity at delivery and 4 outcomes in 309 women with documented *Plasmodium falciparum* infection during follow-up, Benin*

IBA levels at delivery, % inhibition	Placental malaria†			Low birthweight‡			SGA			Preterm birth†	
	OR (95% CI)	p value		OR (95% CI)	p value		OR (95% CI)	p value		OR (95% CI)	p value
Overall sample	NA	NA		n = 290	0.028		n = 264	0.0084		NA	NA
Second quartile, 28–43	NA	NA		0.39 (0.15–1.01)	NA		0.22 (0.08–0.59)	NA		NA	NA
Third quartile, 43–63	NA	NA		0.27 (0.10–0.76)	NA		0.34 (0.13–0.88)§	NA		NA	NA
Higher quartile, >63	NA	NA		0.22 (0.07–0.72)	NA		0.35 (0.13–0.94)§	NA		NA	NA
Women not infected at inclusion (first infection after inclusion)	n = 163	0.0085		NA	NA		NA	NA		n = 173	0.0324
Second quartile, 28–43	0.66 (0.22–2.04)	NA		NA	NA		NA	NA		0.10 (0.01–0.93)§	NA
Third quartile, 43–63	0.19 (0.05–0.72)§	NA		NA	NA		NA	NA		0.09 (0.01–0.89)§	NA
Higher quartile, >63	0.21 (0.06–0.76)§	NA		NA	NA		NA	NA		0.18 (0.03–1.18)	NA
Women infected at inclusion	n = 103	0.604		NA	NA		NA	NA		n = 87	0.969
Second quartile, 28–43	1.29 (0.30–5.50)	NA		NA	NA		NA	NA		0.68 (0.14–3.20)	NA
Third quartile, 43–63	2.02 (0.52–7.91)	NA		NA	NA		NA	NA		0.87 (1.96–3.83)	NA
Higher quartile, >63	0.90 (0.19–4.35)	NA		NA	NA		NA	NA		NA	NA

*SGA, small for gestational age; OR, odds ratio; IBA, inhibition of binding assay; NA, not applicable. Analysis was conducted by using multivariate logistic regression adjusting for center, gravidity, infection at inclusion, and testing the interaction between IBA level and infection at inclusion. †In the absence of a major interaction, results of the model without interaction are shown. ‡In the instance of a major interaction, results were stratified by the presence or absence of infection at inclusion. §When ORs for 2 contiguous quartiles were similar, p values were computed after grouping those quartiles.

## Discussion

Primigravidae have the most severe consequences of pregnancy-associated malaria because they lack specific protective immunity. Specific immune responses are usually initiated during the first pregnancy and result in protection in subsequent pregnancies. Although several studies showed that antibodies against infected erythrocytes in the placenta are associated with improved pregnancy outcomes (*13*,*15*,*32*), the precise mechanisms involved remain to be clarified. Use of vaccination as a new approach to prevent placental infections requires that specificities of these antibodies be determined. The current consensus is that the VAR2CSA protein specifically expressed by placental parasites is the primary target of such antibodies, but the epitopic target(s) within this large protein are yet to be defined.

One noteworthy observation is early appearance of antibodies against infected erythroctyes in the placenta in primigravidae. These women were recruited mainly at the beginning of their second trimester of pregnancy; such antibodies were present in 40%–70%. Similar prevalences among primigravidae were reported in Senegal (*13*) and Cameroon (*33*), which suggests that women are infected with placental-type parasites early in pregnancy, consistent with our previous report of placental-type parasites in pregnant women in the first trimester (*34*). This finding emphasizes the need to prevent *P. falciparum* infection in early pregnancy.

Parasitemia transiently decreased from 16% to 4% during pregnancy and increased to 12% at delivery. This decrease is the result of IPTp because the decrease was centered at intake periods (G. Cottrell, N. Tuikue Ndam, unpub. data). Despite IPTp, 46% of women had parasitemia during the follow-up period, which highlights the need to improve current approaches to prevent pregnancy-associated malaria.

Overall, levels of antibodies against several antigens tended to decrease during pregnancy, including those specific for placental-type parasites. Although plasma volume expansion associated with pregnancy might contribute to this overall decrease in antibody, the decrease observed also reflects a lack of exposure, as shown by the profiles in women who remained uninfected after inclusion. A previous study showed that levels of IgG against VAR2CSA decreased rapidly over a 3-month period in the absence of antigenic stimulation (*13*). In women who were infected and treated only at inclusion, all antibody levels decreased between inclusion and delivery. This finding was not accompanied by their functional capacity to inhibit binding of infected erythrocytes that was maintained, which reiterates the need to distinguish between assay results in the context of vaccine development and emphasizing utility of functional assays.

Conversely, in women infected during the follow-up period, levels of antibody against VAR2CSA increased between inclusion and delivery and were higher for multigravidae than for primigravidae. The binding inhibitory capacity of plasma was also higher in multigravidae. Our study analyzed binding inhibition properties for a large number of pregnant women and corroborated results of earlier studies, which showed parity-dependent acquisition of binding inhibitory capacity of plasma (*13*,*14*).

Multivariate analyses indicated that responses to only a restricted number of VAR2CSA domains could be deemed protective against negative outcomes during pregnancy. DBL3-specific antibodies were associated with reduced rates of infections during pregnancy and placental infection at delivery. In women in Cameroon early during pregnancy, high levels of IgG against multiple VAR2CSA domains were associated with a lower risk for placental infection at delivery (*33*). In our study, high levels of IgG against VAR2CSA early during pregnancy were also related to protection against subsequent infection, which highlights the role of DBL3-specific IgG. In addition, DBL1–DBL2–specific antibodies were associated with a 67% reduction in LBW. This finding emphasizes the key role of the VAR2CSA N-terminal region that contains the minimal CSA binding site (*20*,*21*,*35*,*36*). A recent report suggested that IgG responses to the VAR2CSA minimal binding site was not pregnancy specific and that levels of these antibodies at delivery were not associated with protection from placental infection (*37*), which are in contrast with data from other studies.

Although the design of the study of Babakhanyan et al. (*37*), differed only slightly from that of our study, antigenic constructs were radically different. We used a larger VAR2CSA N-terminal construct (DBL1–DBL2), whereas Babakhanyan et al. used a minimal internal domain (ID1–ID2) construct. The other major difference was the method used to measure antibody. We used an ELISA and optimized conditions for each antigen. However, Babakhanyan et al. used a Luminex (Austin, TX, USA) assay that measures multiple analytes simultaneously in 1 reaction well and fixed default background values, which resulted in difficulties with interpretation of results. Our data highlight the need to clarify the contribution of antibodies to ID1–ID2, one of the current vaccine candidates, in protection against placental malaria. Our data indicate that antibody responses to DBL3X and DBL1–DBL2 represent surrogates of protection against placental malaria.

The functional capacity of antibodies to mediate inhibition of infected erythrocyte adherence to CSA is enhanced after infection and is sustained despite the decrease in levels of antibodies against VAR2CSA. We divided women into various subgroups by history of infection, which showed the pivotal role of infections in increasing quality of antibodies. The rationale for vaccination to prevent placental malaria is that it should induce immune memory against infected erythrocytes in the placenta, lead to an accelerated response at exposure, and limit deleterious effects of infections on pregnancy outcomes. We previously demonstrated that pregnant women might be exposed to placental-type parasites in early pregnancy, exposure increases with gestational age, and that women are more often infected with placental-type parasites later in pregnancy (*38*). These findings might explain why the relative increase in IBA levels was greater for infections after inclusion than in those at inclusion (Figure 3, panel B). This capacity at delivery protects against placental infection, LBW, and PTB. Antibody maturation after natural boosting leads to acquisition of a binding inhibitory property that contributes to clearing or preventing placental infection. The rationale for the association of delivery with LBW, SGA, and PTB, which has complex etiologies, is not as clear as that for placental infection. Previous studies showed that *P. falciparum* infections at delivery are strongly associated with LBW and PTB. We showed that functional antibody response at delivery is associated with the absence of placental infection. Such functional response might prevent placental infections during pregnancy.

Our data support the idea that inducing protective immunity against placental parasites by vaccination requires induction of antibodies that inhibit binding of infected erythrocytes to CSA. We measured the quantity and quality of antibodies in a cohort of pregnant women for whom detailed clinical histories were available. Results provide evidence that support current efforts to develop a subunit vaccine based on VAR2CSA constructs derived from its N-terminal portion.
